# Isosteviol Sensitizes sarcK_ATP_ Channels towards Pinacidil and Potentiates Mitochondrial Uncoupling of Diazoxide in Guinea Pig Ventricular Myocytes

**DOI:** 10.1155/2016/6362812

**Published:** 2016-02-02

**Authors:** Zhuo Fan, Ting Wen, Yaoxu Chen, Lijie Huang, Wei Lin, Chunxia Yin, Wen Tan

**Affiliations:** ^1^School of Bioscience and Bioengineering, South China University of Technology, Guangzhou 510006, China; ^2^Pre-Incubator for Innovative Drugs and Medicine, South China University of Technology, Guangzhou 510006, China; ^3^Guangdong Provincial Key Laboratory of Fermentation and Enzyme Engineering, South China University of Technology, Guangzhou 510006, China

## Abstract

K_ATP_ channel is an important mediator or factor in physiological and pathological metabolic pathway. Activation of K_ATP_ channel has been identified to be a critical step in the cardioprotective mechanism against IR injury. On the other hand, desensitization of the channel to its opener or the metabolic ligand ATP in pathological conditions, like cardiac hypertrophy, would decrease the adaption of myocardium to metabolic stress and is a disadvantage for drug therapy. Isosteviol, obtained by acid hydrolysis of stevioside, has been demonstrated to play a cardioprotective role against diseases of cardiovascular system, like anti-IR injury, antihypertension, antihyperglycemia, and so forth. The present study investigated the effect of isosteviol (STV) on sarcK_ATP_ channel current induced by pinacidil and mitochondrial flavoprotein oxidation induced by diazoxide. Our results showed that preincubating cells with STV not only increased the current amplitude and activating rate of sarcK_ATP_ channels induced by pinacidil but also potentiated diazoxide-elicited oxidation of flavoprotein in mitochondria.

## 1. Introduction

The ATP-sensitive potassium (K_ATP_) channel was first discovered by Noma in 1983 in isolated membrane patches prepared from guinea pig ventricular myocytes and subsequently identified in many other tissues, including brain, smooth muscle, skeletal muscle, and pancreas [1]. These channels are inhibited by intracellular ATP and activated by intracellular nucleoside diphosphates, thereby coupling cell metabolic condition to membrane electrical activity in various cell types [2, 3]. Structurally, K_ATP_ channels are heterooctamers composed of four Kir6.x and four sulfonylurea receptor (SUR) subunits [4]. Kir6.x subunits form the K^+^ transporting channel pore, whereas SURs serve as regulatory subunits and endow the channel with sensitivity to sulfonylureas, nucleotides, and the K_ATP_ channel openers such as pinacidil and diazoxide [5, 6]. Functionally, K_ATP_ channels have been demonstrated to be involved in many physiology activities like insulin secretion, smooth muscle contraction, and so forth [7–9].

Opening of K_ATP_ channel has been shown to be an endogenous protective mechanism in response to various stresses under altered metabolic states, including hyperglycemia, hypertension, ischemia, and hypoxia. For example, prepharmacological opening of K_ATP_ channels has been demonstrated to play a cardioprotective role against IR injury and K_ATP_ channel has been identified to be a key component in the phenomenon termed ischemic preconditioning (IPC), in which single or multiple brief periods of ischemia have been shown to protect the heart against a subsequent prolonged ischemic insult [10]. Both sarcolemmal (sarcK_ATP_) and mitochondrial K_ATP_ (mitoK_ATP_) channels have been proposed to be involved in the cardioprotective role against IR injury through distinct mechanism [11–14]. ATP-sensitive K channels are essential for maintaining the cellular homeostasis against various metabolic stresses. Disease induced structural remodeling of cardiomyocytes may decrease or diminish the sensitivity of K_ATP_ channel to ATP and/or its openers and alter this adaptive response to such stresses [15–18]. The dysfunctional K_ATP_ channels in these conditions may fail to protect the myocardium from metabolic stresses or make the pharmacological therapy targeted to K_ATP_ channels ineffective. So, finding a drug or substance which would increase the sensitivity but not induce direct activation of K_ATP_ channel is necessary.

Isosteviol is a derivative of stevioside, which has been used commercially as a sugar substitute for years [19]. Studies indicated that both stevioside and isosteviol may possess a variety of biological activities including antihypertension, antihyperglycemia, anti-inflammatory, and potential antitumor effects [20–26]. Besides, the myocardium protective effects of isosteviol against ischemia-reperfusion injury have been reported by Tan and other groups [27–29]. According these studies, isosteviol could reduce the infraction area and restore the contractility in cardiac IR in vivo and in isolated hearts without introducing or even improving arrhythmia [27–29], which as an adverse effect limits the usage of classical KCOs in clinic [30]. The protective effects of isosteviol could be partially blocked by 5-HD, a mitoK_ATP_ channel blocker, which suggested a potential involvement of mitoK_ATP_ in the protective mechanism of isosteviol [27–29]. Despite that, the underlying mechanism of isosteviol against ischemia and other stresses at molecular level is not clear so far and needs to be further investigated.

The present study investigated the possible effects of isosteviol sodium (the sodium salt of isosteviol, STV) on sarcK_ATP_ currents induced by pinacidil and flavoprotein fluorescence elicited by diazoxide in isolated guinea pig cardiomyocytes. We found that isosteviol potentiated both the pinacidil-induced sarcK_ATP_ channel current and diazoxide-elicited flavoprotein oxidation, but surprisingly isosteviol sodium alone played no effects on either of them. In addition, the potentiation effects of STV were completely blocked by N-acetyl-cysteine (NAC)—a ROS scavenger. Since the flavoprotein oxidation induced by diazoxide is often used to investigate the activity of mitoK_ATP_ channel as an indirect approach on intact cell [31], we infer from the results that isosteviol may act as a sensitizer or modulator but not a direct opener for both sarc- and mitoK_ATP_ channels and the sensitization effects of STV on both channels are ROS dependent.

## 2. Materials and Methods

### 2.1. Isolation of Guinea Pig Ventricular Myocytes

Single ventricular myocytes were isolated from guinea pig hearts using a standard enzymatic technique. The protocols were approved by our institutional ethics committee. Briefly, adult guinea pigs (250–350 g) were anesthetized with an injection of 5% pentobarbital sodium (0.2 mL/100 g). Heparin (500 U/Kg) was administered to prevent coagulation during heart removal. Then, hearts were rapidly removed, mounted on a Langendorff perfusion apparatus, and retrogradely perfused with Ca^2+^-free tyrode solution composed of (in mM) NaCl 120, KCl 5.4, MgCl_2_ 0.5, HEPES 25, and glucose 10 (pH 7.4 with NaOH) at 37°C. After 5 min, the perfusion solution was changed to the tyrode solution containing type II collagenase (0.6 mg/mL, Sigma) and Ca^2+^ (50 *μ*M) for more than 30 mins. After perfusion, the ventricular tissue was cut into small pieces in a petri dish with the same solution and was shaken gently for the dispersion of dissociated cardiac myocytes. A 250 *μ*M mesh screen was used to separate the isolated cardiac myocytes from cardiac tissue. Then, cells were collected by centrifugation at 500 rpm for 45 s. Finally, the Ca^2+^ concentration was gradually restored to 1 mM. Cells were stored in M199 medium at a 37°C CO_2_ incubator until use.

### 2.2. Electrophysiological Recordings

Whole-cell patch-clamp recordings were performed at room temperature with an EPC-10 amplifier (HEKA, Lambrecht, Germany). Cells were placed in an experimental chamber mounted on a stage of an inverted microscope (Nikon, Japan). Pipettes were pulled from borosilicate glass (Sutter, Novato, CA) and had a resistance of between 2 and 4 MΩ. The bath perfusate for sarcK_ATP_ channel and AP recording was the Ca^2+^ containing tyrode's solution consisting of (in mM) NaCl 137, KCl 5.4, MgCl_2_ 1.2, CaCl_2_ 1, NaH_2_PO_4_ 1.2, HEPES 20, and glucose 10, with the pH controlled to 7.4 with NaOH. The pipette solution was (in mM) KCl 140, MgCl_2_ 2, CaCl_2_ 1, EGTA 11, Na_2_ATP 1, and HEPES 10 (pH 7.2, with KOH). K_ATP_ channel opener pinacidil and antagonist glibenclamide were added to the bath with a perfusion drug delivery system (Scientific Instruments, Cambridgeshire, UK). The pipette solution for *I*_Kr_ was (in mM) KCl 135, MgCl_2_ 2, HEPES 10, EGTA 10, and Mg-ATP 5 (pH 7.2 with KOH). Nifedipine (10 *μ*M) and chromanol B (10 *μ*M) were added to the bath solution to block L-type calcium and *I*_Ks_ currents, respectively. For L-type calcium channel recording, the bath solution was (in mM) TEA-Cl 135, CaCl_2_ 2, glucose 10, HEPES 10, and MgCl_2_ 1. The internal solution was (in mM) CsCl 110, Mg-ATP 5, EGTA 10, and TEA-Cl 30 (pH 7.2 with CsOH). STV was delivered by preincubating cells in the drug containing culture solution for record of currents or by a drug delivery system for AP recording. For investigating the role of ROS on the potentiation effects of STV, 100 *μ*M reactive oxygen species scavenger NAC was coadministered with STV.

K_ATP_ channel currents were elicited by applying the opener pinacidil at a concentration of 100 *μ*M and the protocol used was a depolarizing voltage step to 0 mV for 500 ms from a holding potential of −40 mV applied every 30 s. *I*-*V* curves of K_ATP_ currents were obtained by applying the voltage steps between −80 and +60 mV with a 10 mV increment. In order to compensate for variations in cell size, currents were normalized to cell capacitance and reported as current densities (pA/pF). APs were measured at the current-clamp mode and evoked by applying a 5-ms depolarizing square pulse with the amplitude of 800 pA. Action potential duration was measured at 90% repolarization (APD_90_). *I*_Kr_ currents were elicited by applying the voltage steps from −40 to +40 mV for 1 s and then repolarized to −40 mV for 1 s for tail current recording. The L-type calcium currents were evoked by step depolarization to test potentials between −40 mV and +60 mV for 500 ms. Data were sampled at 10 kHz and filtered at 2.9 kHz. Cell capacitance (*C*_*m*_) and series resistance (*R*_*s*_) were electrically compensated in the voltage clamp experiments. The PatchMaster (HEKA, Lambrecht, Germany) and origin 8 software (OriginLab, Northampton, MA) were used for data acquisition and analysis, respectively.

### 2.3. Flavoprotein Fluorescence Measurements

Opening of mitoK_ATP_ channels dissipates the inner mitochondrial membrane potential established by the proton pump, which accelerates electron transfer by the respiratory chain and leads to net oxidation of the mitochondria. Mitochondrial redox state can be monitored by recording the fluorescence of flavoprotein in the mitochondria as described by Liu et al. [31]. Exposure to dinitrophenol (DNP) uncouples respiration from ATP synthesis, collapses the mitochondrial potential, and induces maximal oxidation. The values of flavoprotein fluorescence were expressed as a percentage of the DNP-induced fluorescence. The fluorescence intensity with the test substances was calculated according to the following equation:

(1) $$\begin{array}{c} Percentage\;\;I_{f}=\frac{I_{f}-I_{bas}}{I_{DNP}-I_{bas}}*100\%, \end{array}$$

where *I*_*f*_, *I*_DNP_, and *I*_bas_ were the fluorescence intensity during exposure to the test agent (diazoxide or isosteviol), DNP, and baseline, respectively. Images of endogenous flavoprotein fluorescence were obtained with a Zeiss 710 confocal laser-scanning microscope (ZEISS, Germany). Fluorescence was excited by the 488-nm line of an argon laser, and the emission at 530 nm was recorded. A time series of images were collected at intervals of 10 seconds. Images were analyzed on a personal computer with the software ZEN2011 (ZEISS, Germany). The extracellular solution was Hanks' balanced salts solution (HBSS, Sigma). STV was delivered after diazoxide or by incubating cells with it previously. The incubation period was more than 1 hr, which is consistent with that needed to observe the maximal effect of STV for sarcK_ATP_ current. For investigating the effect of ROS, 100 *μ*M NAC was coadministered with STV. All the recordings were performed at 37°C.

### 2.4. Chemicals and Drugs

The sodium salt of isosteviol was provided by Key Pharma Biomedical Inc. All chemicals for tyrode's solution and external and internal solution of patch-clamp recording were bought from Sigma Chemical Co. Collagenase was purchased from Worthington. Diazoxide, pinacidil, glibenclamide, and DNP were also purchased from Sigma Chemical Co. and dissolved in DMSO before being added to experimental solutions. The final concentration of DMSO was ≤0.1%. Pentobarbital sodium was obtained from Sangon Biotech (Shanghai) and heparin was obtained from Aladdin Reagent.

### 2.5. Statistical Analysis

The data were expressed as mean ± SEM (standard error of the mean), *n* = number of independent experiments. Statistical analysis was performed by one-way ANOVA using a Bonferroni test. Significant differences between groups were defined at ^*∗*^ *p* < 0.05, ^*∗∗*^ *p* < 0.01.

## 3. Results

### 3.1. SarcK_ATP_ Channel Was Elicited by Pinacidil and Blocked by Glibenclamide

**Figure 1:**
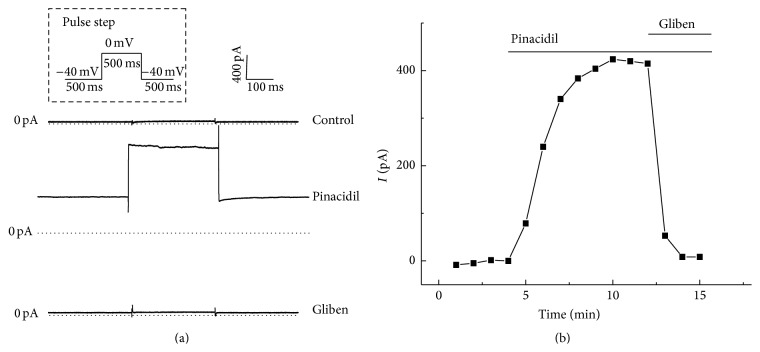
SarcK_ATP_ channel current was elicited by pinacidil and blocked by glibenclamide. (a) Representative whole-cell current traces of sarcK_ATP_ channel without pinacidil (up), with 100 *μ*M pinacidil (middle) and with 10 *μ*M glibenclamide (bottom). (b) The representative time course of K_ATP_ channel activation by pinacidil and inhibition by glibenclamide.

SarcK_ATP_ channel current was elicited by a specific potassium channel opener, pinacidil (100 *μ*M), measuring at a 500 ms test pulse of 0 mV from a holding potential of −40 mV.  Figure 1(a) shows the representative trace of whole-cell *I*_K_ATP__ activated by pinacidil (Figure 1(a) middle). Spontaneous activation of *I*_K_ATP__ was not observed in the absence of pinacidil during control condition (Figure 1(a) top). The outward current was completely suppressed by the antagonist glibenclamide (1 *μ*M, Figure 1(a) bottom), which indicates that the pinacidil-elicited current is caused by the activation of sarcolemmal K_ATP_ channels. The time course of K_ATP_ channel activation by pinacidil is shown in Figure 1(b). The current was usually activated within 5 minutes after pinacidil application. Current amplitude was measured at the end of the test pulse to 0 mV.

### 3.2. STV-Preincubation Increased the Current Density and Activating Rate of SarcK_ATP_ Channel Elicited by Pinacidil

**Figure 2:**
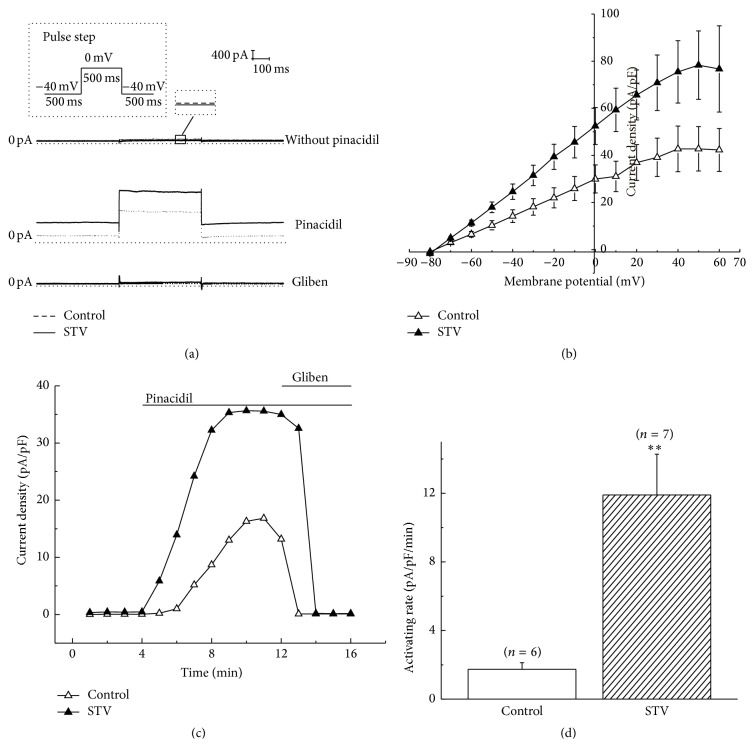
STV increased the amplitude and activating rate of pinacidil-induced K_ATP_ current. (a) Representative pinacidil-induced K_ATP_ currents of STV-preincubated (10 *μ*M) and control group. (b) Effect of 10 *μ*M STV on current-voltage (*IV*) relationship of pinacidil-induced K_ATP_ channel current. Currents were normalized to cell capacitance. (c) The time course of K_ATP_ channel activation by pinacidil for STV-preincubated and control group. (d) Comparison of activation rates of K_ATP_ channel between STV-preincubated and control group. *∗∗* means *p* < 0.01.

Next, we investigated the effect of isosteviol sodium (STV) on sarcK_ATP_ channel current by preincubating the isolated myocytes with the drug. We found that STV alone did not elicit K_ATP_ channel current at the concentration of 10 *μ*M (Figure 2(a) up), whereas preincubating cells with STV of the same concentration significantly increased K_ATP_ channel current elicited by 100 *μ*M pinacidil compared with control group (Figure 2(a) middle). 1 *μ*M glibenclamide blocked both the currents of STV-preincubation and control group (Figure 2(a) bottom). At 0 mV, the current density of K_ATP_ channels was increased to 52.5 ± 7.8 pA/pF (*n* = 7, *p* < 0.05) by preincubating cells with 10 *μ*M STV from 30.0 ± 6.0 pA/pF (*n* = 6) of control condition. To investigate the effect of STV on current-voltage relationship of the K_ATP_ current induced by pinacidil, command voltage pulses of 500 ms in duration to various membrane potentials (from −80 mV to +60 mV) were applied to the ventricular myocytes. Data shows that STV increased the current density of pinacidil-elicited K_ATP_ channel at all test potentials above −70 mV (Figure 2(b)). The reversal potential of K_ATP_ channel was not changed by STV. Both had a reversal potential of about −80 mV, which is close to the estimated equilibrium potential for K^+^ under the experimental conditions. Besides current amplitude, the activating rate of pinacidil-induced sarcK_ATP_ current was also examined. Activating rate was calculated by dividing the pinacidil-elicited current density changes by the time consumed.  Figure 2(c) represents the activation course of sarcK_ATP_ channel induced by pinacidil of control group and drug-preincubated group. Data shows that the activating rate of STV-preincubated group was significantly increased compared with control group. The activating rates were 11.9 ± 2.4 (*n* = 7) and 1.7 ± 0.4 (*n* = 6) pA/pF/min for drug-preincubated and control group, respectively (Figure 2(d)).

### 3.3. The Time and Concentration Dependence of STV on Pinacidil-Activated K_ATP_ Current

**Figure 3:**
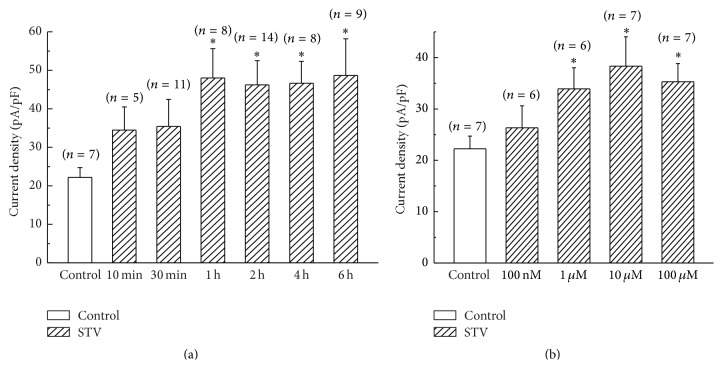
Time and concentration dependence of STV on pinacidil-activated K_ATP_ current. (a) The time dependence of STV-preincubation (10 *μ*M) on pinacidil-induced K_ATP_ channel current. (b) The concentration dependence of STV on K_ATP_ current with incubation period of more than 1 hr. *∗* means *p* < 0.05.

To investigate the time dependence of STV on pinacidil-induced sarcK_ATP_ currents, we incubated the isolated myocytes in STV-containing tyrode solution with different time at the concentration of 10 *μ*M.  Figure 3(a) indicates that the current density of *I*_K_ATP__ was gradually increased with the incubation time prolonged and got saturated after 1 hour. The current densities of *I*_K_ATP__ were 48.0 ± 7.6 (*n* = 8), 46.2 ± 6.3 (*n* = 14), 46.6 ± 5.7 (*n* = 8), and 48.7 ± 9.5 (*n* = 9) pA/pF at 1, 2, 4, and 6 hrs, respectively, which were significantly larger than that of 22.2 ± 2.5 pA/pF (*n* = 7) for control group. The concentration dependence of STV on pinacidil-activated K_ATP_ current was also investigated by preincubating cells with STV for more than 1 hour with different concentration.  Figure 3(b) shows that the concentration of 1 *μ*M or more of STV significantly increased the current density of *I*_K_ATP__. The current densities of K_ATP_ channel elicited by pinacidil were 33.9 ± 4.1 (*n* = 6) and 38.3 ± 5.7 (*n* = 7) pA/pF at the concentration of 1 *μ*M and 10 *μ*M, respectively, both of which were significantly larger than that of control group (22.2 ± 2.5 pA/pF, *p* < 0.05). 100 *μ*M STV did not further enlarge the current density of *I*_K_ATP__ compared with 10 *μ*M.

### 3.4. Effect of STV on I_Kr_ and L-Type Ca^2+^ Current

**Figure 4:**
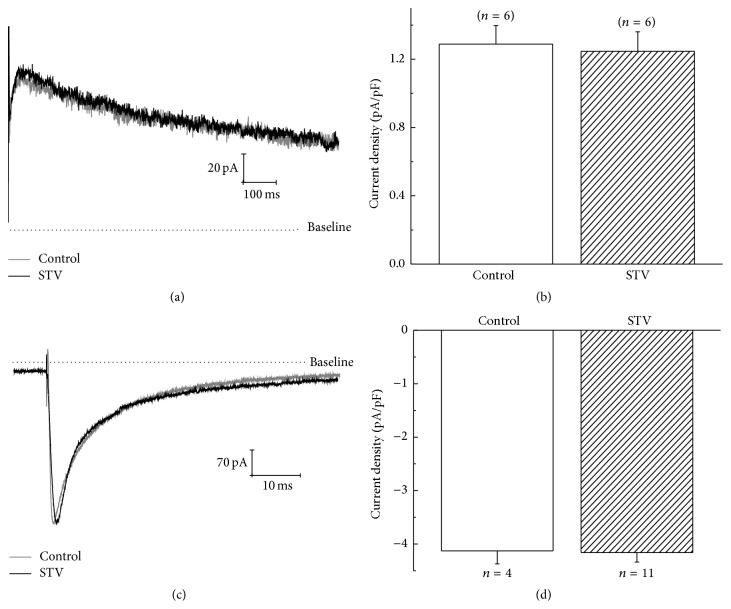
Effects of STV on *I*_Kr_ and L-type calcium current of guinea pig ventricular myocytes. (a) and (b) show the representative currents and the mean current densities of *I*_Kr_ tail current at +40 mV for STV-preincubated and control group. (c) and (d) show the representative currents and mean current density of L-type calcium current at +10 mV for STV-preincubated and control group, respectively. Cells were incubated with STV for no less than 1 hour before *I*_Kr_ and *I*_CaL_ recordings.

Besides K_ATP_ channel, we also examined the effect of STV on the rapidly activated delayed rectifier K^+^ (*I*_Kr_) and L-type Ca^2+^ channel (*I*_CaL_) currents. Cells were incubated with STV for no less than 1 hour before recording. Figures 4(a) and 4(b) show that the tail current of *I*_Kr_ at +40 mV was not affected significantly by preincubating cells with 10 *μ*M STV. The mean current densities of *I*_Kr_ tail currents at +40 mV for drug-preincubated and control group were 1.2 ± 0.1 and 1.3 ± 0.1 pA/pF, respectively. Figures 4(c) and 4(d) show that the current amplitude of L-type calcium channel at +10 mV was not affected by STV either. The current densities of calcium channel at +10 mV were 4.2 ± 0.2 and 4.1 ± 0.2 pA/pF for drug-preincubated and control group, respectively. Neither the amplitude of *I*_Kr_ nor *I*_CaL_ was affected by STV-preincubation.

### 3.5. Effect of STV on Action Potential of Guinea Pig Ventricular Myocytes

**Figure 5:**
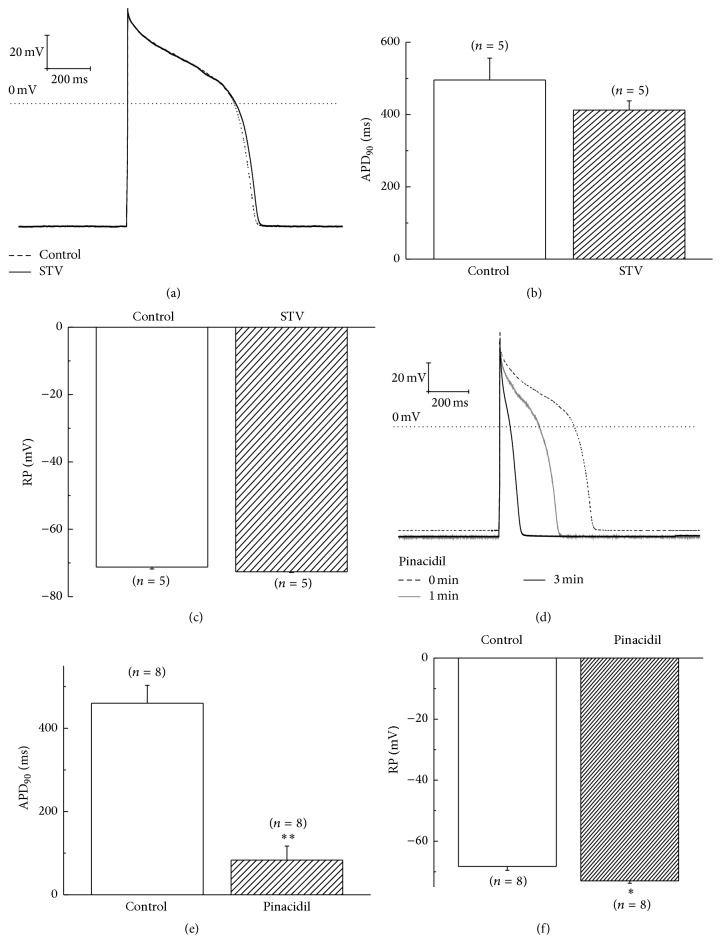
Effects of STV and pinacidil on action potential of guinea pig ventricular myocytes. (a) Representative AP curve of STV-preincubated and control ventricular myocytes. (b) and (c) Effects of STV on action potential duration at 90% of repolarization (APD_90_) and resting membrane potential (RMP), respectively. (d) Effect of pinacidil on action potential of ventricular myocytes. (e) and (f) Effects of pinacidil on action potential duration at APD_90_ and RMP, respectively. *∗* means *p* < 0.05 and *∗∗* means *p* < 0.01.

Besides the effects of STV on membrane currents, we also investigated its effect on action potential of guinea pig ventricular myocytes. STV was applied by a perfusion drug delivery system. We observed the change of the action potential duration (APD) and the resting membrane potential (RMP) after 5 mins of drug application, when the action potential of cardiomyocytes becomes steady. Data show that 10 *μ*M STV changed neither the action potential duration (APD_90_) nor the resting membrane potential (RMP) of ventricular myocytes significantly (Figures 5(a), 5(b), and 5(c)). The APD and RMP for control group were 495.5 ± 60.5 ms (*n* = 5) and −71.2 ± 0.6 mV (*n* = 5), which were not significantly different from the value of 412.4 ± 25.6 ms and −72.6 ± 0.2 mV for STV group. These results are consistent with the above results that STV alone had no effect on sarcK_ATP_, *I*_Kr_, and L-type calcium currents. For comparison, we also examined the effect of pinacidil on action potential of myocytes. Figures 5(d), 5(e), and 5(f) show that both the APD_90_ and RMP of cardiomyocytes were significantly affected by pinacidil. The APD_90_ was shortened from 460.3 ± 42.9 ms to 83.2 ± 33.9 ms (*n* = 8, *p* < 0.01), whereas the RMP was hyperpolarized from −68.3 ± 1.2 mV to −73 ± 0.8 mV (*n* = 8, *p* < 0.05) in about 3 mins by pinacidil's application. We also investigated the preincubation effect of STV on pinacidil-induced shortage of AP. The results showed that STV-preincubation made the shortening of AP by pinacidil faster, although the change did not reach a significant level (data are not shown). We probably could attribute this unexpected result to the extremely rapid influence of pinacidil on AP and the state of cardiomyocytes, which often causes cells to shrink and die in several minutes.

### 3.6. STV-Preincubation Enhanced the Flavoprotein Fluorescence Induced by Diazoxide

**Figure 6:**
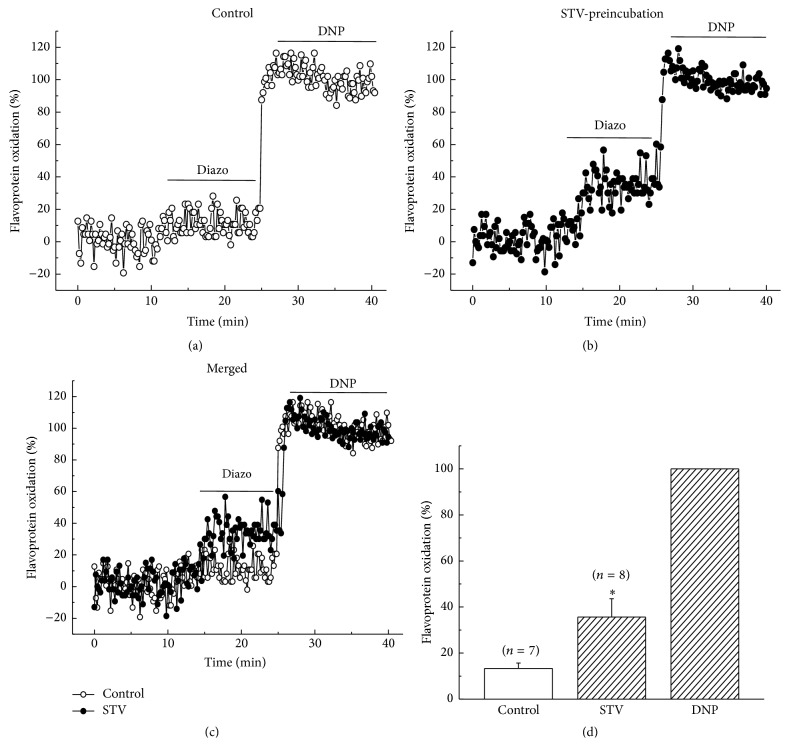
Effect of STV-preincubation on fluorescence intensity of flavoprotein induced by diazoxide. (a) and (b) Time course of flavoprotein fluorescence induced by diazoxide for control and STV-preincubation group. (c) A merged figure from (a) and (b). (d) The mean values of flavoprotein fluorescence normalized by DNP of control and STV-preincubation group. The preincubation time was no less than 1 hour.

Diazoxide, as a specific mitoK_ATP_ channel opener, is usually used to investigate mitoK_ATP_ channel activity pharmacologically. Mitochondrial flavoprotein fluorescence was measured during exposure to diazoxide (200 *μ*M) and the percentage to DNP (200 *μ*M) induced fluorescence was calculated for comparison.  Figure 6 showed that preincubating cells with 10 *μ*M STV significantly enhanced the intensity of diazoxide-induced flavoprotein fluorescence. The normalized fluorescence intensity was 35.6 ± 8.0% for STV-preincubated group, which was about 2.5 times larger than control group (13.4 ± 2.4%) (*n* = 6, *p* < 0.05, Figure 6(c)). STV alone did not induce an obvious flavoprotein fluorescence compared with baseline (Figure 6(b), 0–10 min).

### 3.7. STV Had No Effect on Flavoprotein Oxidation When Applied after Diazoxide

**Figure 7:**
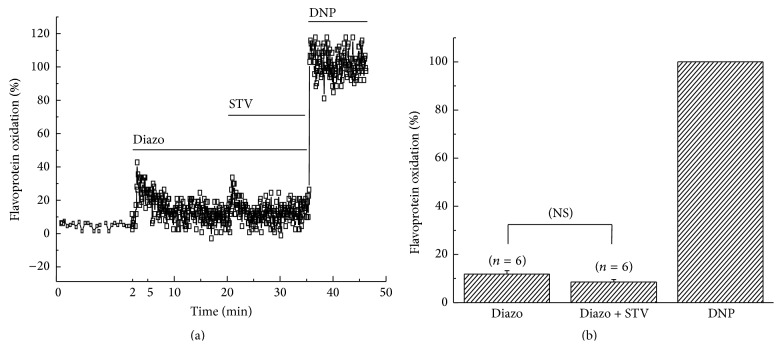
Effect of STV on flavoprotein oxidation applied after diazoxide. (a) Time course of flavoprotein fluorescence induced by diazoxide (Diazo) and Diazo + STV subsequently. The first 2 minutes-duration was elongated to get a good view of baseline. (b) Normalized mean values of fluorescence intensity caused by Diazo and Diazo + STV, respectively.

In addition, we also investigated the effect of STV-application after diazoxide. Figures 7(a) and 7(b) showed that 10 *μ*M STV-application after diazoxide did not induce a significant change in the fluorescence intensity of flavoprotein either. The percentage oxidations induced by diazoxide and diazoxide + STV were 11.8 ± 1.4% and 8.5 ± 1.1%, respectively (*n* = 6). These results suggest that STV had no influences on the uncoupling effect of diazoxide when applied after diazoxide.

### 3.8. The Potentiation Effects of STV on SarcK_ATP_ Current and Flavoprotein Oxidation Were ROS Dependent

**Figure 8:**
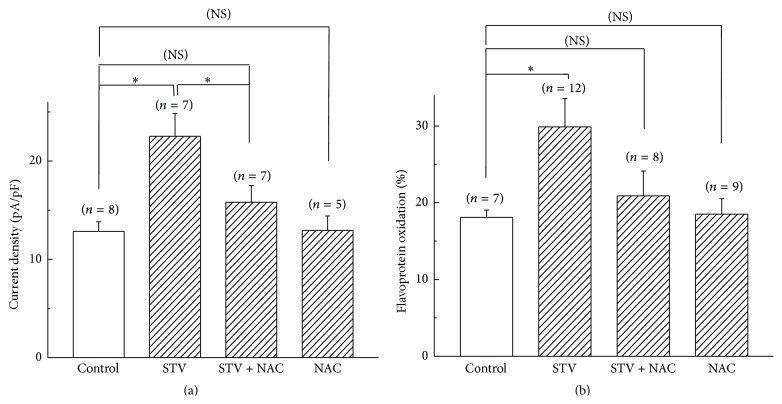
NAC blocked the potentiation effects of STV on sarc- and mitoK_ATP_ channels. (a) Current density of *I*_sarcK_ATP__ elicited by pinacidil for control, STV-preincubation, STV + NAC, and NAC group. (b) The DNP normalized fluorescence intensity induced by diazoxide for control, STV-preincubation, STV + NAC, and NAC group. *∗* means *p* < 0.05 and NS means no significant difference.

To examine if the potentiation effects exerted by STV on sarcK_ATP_ channels and mitochondrial flavoprotein oxidation were mediated by ROS, the ROS scavenger NAC was coadministered.  Figure 8(a) shows that preincubation of cardiomyocytes with 10 *μ*M STV significantly increased the current density elicited by 50 *μ*M pinacidil (22.5 ± 2.3 pA/pF (*n* = 7) for STV group versus 12.8 ± 1.0 pA/pF (*n* = 8) for control group, *p* < 0.05), whereas coadministering with 100 *μ*M NAC blocked the enhancing effect of STV (15.8 ± 1.7 pA/pF (*n* = 7) for STV + NAC group). NAC (100 *μ*M) alone had no effects on pinacidil-elicited sarcK_ATP_ channel current. For mitochondria, NAC also blocked the potentiation effect of STV on diazoxide-elicited flavoprotein fluorescence.  Figure 8(b) shows that the percentage oxidations to DNP induced by 200 *μ*M diazoxide were 18.1 ± 1.0%, 29.9 ± 3.7%, and 20.9 ± 3.2% for control, STV, and STV + NAC group, respectively. Similarly, NAC (100 *μ*M) alone had no effect on the fluorescence intensity of flavoprotein elicited by diazoxide (18.5 ± 2.0% for NAC alone group).

## 4. Discussion

The present study investigated the cardioprotective mechanism of isosteviol by examining the effect of isosteviol sodium on sarcolemmal K_ATP_ channel and mitochondrial flavoprotein oxidation on guinea pig ventricular myocytes. For sarcK_ATP_, isosteviol enhanced both pinacidil-activated *I*_K_ATP__ current amplitude and the rate of activation. The effect of isosteviol on sarcK_ATP_ channel currents was time and dose dependent. The longer period or larger dose of incubation with isosteviol resulted in stronger enhancement of K_ATP_ currents. Similarly, in mitochondria, preincubating cells with isosteviol sodium significantly enhanced diazoxide-induced mitochondrial uncoupling indicated by mitochondrial flavoprotein fluorescence, which was not elicited by isosteviol alone. The ROS scavenger NAC prevented STV induced potentiation of both sarcK_ATP_ activity and mitochondrial oxidation. If the diazoxide-induced flavoprotein oxidation could be attributed to mitoK_ATP_ channel activity, then these results indicate that isosteviol, as a modulator but not a direct opener, sensitizes both sarc- and mitoK_ATP_ channels to their openers and the effects of STV on K_ATP_ channels are ROS dependent. Furthermore, the result that STV applied after diazoxide induced no effect on mitoK_ATP_ channel activity suggests that mitoK_ATP_ channels may not respond to isosteviol during activation.

ATP-sensitive potassium channels are thought to provide mechanisms for adaptation of cardiac myocytes to various stresses, including hypoxia, ischemia, hypertensive, and hypertrophy. The cardioprotective role of K_ATP_ channel opening against IR injury by pharmacological method or IPC has been extensively studied. Both sarc- and mitoK_ATP_ channels are demonstrated to be involved. Opening of sarcK_ATP_ channels produced by hypoxia, ischemia, or pharmacological K_ATP_ openers would hyperpolarize cell membrane, shorten action potential duration, inhibit calcium influx, and finally lead to a cardioprotective effect by depression of contractility [11–13]. Opening of mitoK_ATP_ channels could also protect the heart against ischemia-reperfusion (IR) injury by regulating mitochondria matrix volume and maintaining or enhancing ATP synthesis [14]. However, in metabolic stress-induced pathological conditions, the sensitivity of K_ATP_ channel to the metabolic ligand ATP or its pharmacological openers is dramatically diminished. For example, in a model of heart failure induced by transgenic expression of the cytokine tumor factor alpha (TNF*α*), the recognition of the ligand ATP for K_ATP_ channels was significantly impaired [15]. Another study showed that the response of K_ATP_ channel to its opener was markedly diminished for hypertensive rats [16]. Myocardial pathological hypertrophy not only reduces the responsiveness of K_ATP_ channel to ATP but also makes K_ATP_ channel fail to sensitize the opener cromakalim [17, 18]. The impaired responsiveness of K_ATP_ channel to the metabolic ligand or KCOs disrupts K_ATP_ channel mediated cellular stress tolerance. The results of this study showed that isosteviol elevated the sensitivity of both sarc- and mitoK_ATP_ channels to their respective openers, which suggests a potential value of STV in treating stress-induced diseases like hypertrophy or heart failure. In another hand, isosteviol itself did not activate these channels directly like other K_ATP_ channel openers. This is an advantage over classic KCOs which could open K_ATP_ channel directly and are often arrhythmogenic as a consequence. The result that isosteviol did not induce action potential shortening as pinacidil did further confirmed the nonarrhythmogenic property of isosteviol.

Reactive oxygen species (ROS) are a kind of free radical in the living organisms and the main source of them is assumed to be mitochondria [32–34]. Originally, ROS were considered to be the by-products of normal physiological processes and have only destructive roles to the organism metabolism. Recent studies tend to support that moderate increase in ROS plays a critical second messenger role in a variety of physiological functions. In cardiomyocytes, it has been demonstrated that a small increase of ROS level is essential for activating the signaling pathways in IPC and leads to cardioprotection against IR injury [35, 36]. Studies have demonstrated that in the mechanism of IP protection, opening of mitoK_ATP_ channels increased the ROS production, which phosphorylation dependently feed back to K_ATP_ channel again and make the channel open persistently [37]. The results of this study consistently showed that the sensitization effects of STV on sarc- and mitoK_ATP_ channels are ROS dependent. However, the specific molecular mechanism and signaling pathway still remain unclear. For signal transduction of STV to mitochondria, cGMP and PKG may serve as critical factors, considering that almost all signaling cascades for ischemic or pharmacological conditions start from GPCR of sarcolemma and use cGMP as a second messenger and PKG as the terminal kinase interacting with mitochondria [37]. PKC may function as a vital signaling molecular for the signal transduction in mitochondria. Studies demonstrated that in the IP protection cascade mitoK_ATP_ channel opening causes an increase in ROS production, which activates protein kinase C (PKC*ε*), which prolongs the phosphorylation-dependent open state of mitoK_ATP_ by forming a positive feedback loop [37]. For sarcK_ATP_ channels, the mechanism by which STV potentiates the channel activity via a ROS dependent manner is also not well known. Sukhodub et al. in their study identified that AMP-activated kinase (AMPK) is essential for preconditioning-induced recruitment of sarcolemmal K_ATP_ channels [38]. Besides, several investigations have suggested that PKC is involved in controlling the distribution and maintaining an elevated sarcK_ATP_ channel concentration [39, 40]. We speculate, in this study, that AMPK and PKC may be also involved in the ROS dependent potentiation effect of STV on sarcK_ATP_ channels by regulating the trafficking and expression of the channel on sarcolemma.

Since the isolated mitochondria or mitoplasts are unavoidably contaminated with plasma membranes, many studies on mitoK_ATP_ channels tend to use indirect approaches on intact cells by measuring flavoprotein fluorescence, swelling-induced light scatter, or changes of mitochondrial membrane potential. In this study, we investigate the change of flavoprotein fluorescence induced by diazoxide and/or STV and attributed it to the activity of mitoK_ATP_ channels. However, this is a little arbitrary. In fact, the exact molecular composition of mitoK_ATP_ channel remains elusive and the mitochondrial pathways of cardioprotective mechanism have not been clearly understood. Evidence for the existence of a mitochondrial K_ATP_ channel and its cardioprotective role is largely pharmacological. Diazoxide and 5-HD, which are used as selective mitoK_ATP_ opener and blocker, have been criticized due to the lack of specificity and several off-target effects. For example, diazoxide has been identified to inhibit mitochondrial respiratory complex II (succinate dehydrogenase) [41]. Furthermore, the specificity of diazoxide has been questioned, since it could activate sarcK_ATP_ channel at higher concentration [42]. However, these evidences could not absolutely exclude the existence of mitoK_ATP_ channel. Unlike their counterparts in the sarcolemma, which are composed of Kir6.xs and SURs, mitoK_ATP_ channel may be a multiprotein complex containing complex II (succinate dehydrogenase) as an important regulator or component. Inhibition of complex II by diazoxide or other inhibitors may activate mitoK_ATP_ channels [43, 44]. As a supplement, we measured the effect of diazoxide with different concentrations on mitochondria membrane potential. The results showed that although 100 *μ*M diazoxide did not cause a change, concentration of 200 and 300 *μ*M did induce mitochondrial membrane depolarization (data are not shown). This complementary experiment may indicate that the diazoxide-induced flavoprotein oxidation is caused by mitochondrial membrane potential depolarization through probable potassium ion transport. However, this conclusion may be also arbitrary considering that diazoxide could also act as a protonophore and depolarize mitochondria by facilitating transmembrane proton translocation [45].

From this study we still cannot get a clue about how isosteviol sensitizes sarc- or mitoK_ATP_ channels. It is possible that STV may slightly compromise mitochondrial functions and disturb overall cellular energies, which increased the susceptibility of mitochondrial to uncoupling (induced by diazoxide) and elevated the sensitivity of sarcK_ATP_ channels towards KCOs (pinacidil) due to a decreased intracellular ATP/ADP ratio. This speculation would be consistent with the effects of prolonged incubation of cardiomyocytes with STV (Figure 3) and the absence of an acute effect of this drug on flavoprotein fluorescence following diazoxide application (Figure 7). Bienengraeber and his coworkers suggested in their study that SUR of K_ATP_ channel has the intrinsic ATPase activity and KCOs could increase the open probability of K_ATP_ channel by binding to SUR and promoting its ATPase activity [46]. So, the STV may also sensitize K_ATP_ channels by playing a role in the ATPase activity of SURs.

In conclusion, the present study demonstrates that isosteviol sodium could modulate sarcK_ATP_ channel by increasing the pinacidil-elicited transmembrane current. It may also, although not absolutely, potentiate the mitoK_ATP_ channel activity. These potentiation effects of STV on sarc- and mitoK_ATP_ channels are ROS dependent. However, the signal pathway in which isosteviol modulates sarc- or mitoK_ATP_ channel is not clear from this study and merits further investigation. Furthermore, we only studied the sensitization effects of STV on cardiomyocytes in physiological state. Whether or not STV would increase the sensitivity of K_ATP_ channel impaired in pathological conditions needs to be further investigated.

## Abbreviations

SUR: Sulfonylurea receptor
IPC: Ischemic preconditioning
IR: Ischemia-reperfusion
KCO: Potassium channel opener
5-HD: 5-Hydroxydecanoate
ROS: Reactive oxygen species
STV: Isosteviol
NAC: N-Acetyl-cysteine
APD: Action potential duration
DNP: Dinitrophenol
RMP: Resting membrane potential
PKC: Protein kinase C
AMPA: AMP-activated kinase.

## References

[1] Noma A. (1983). ATP-regulated K^+^ channels in cardiac muscle. *Nature*.

[2] Ashcroft F. M. (1988). Adenosine 5′-triphosphate-sensitive potassium channels. *Annual Review of Neuroscience*.

[3] Terzic A., Jahangir A., Kurachi Y. (1995). Cardiac ATP-sensitive K^+^ channels: regulation by intracellular nucleotides and K^+^ channel-opening drugs. *The American Journal of Physiology—Cell Physiology*.

[4] Inagaki N., Gonoi T., Clement J. P. (1995). Reconstitution of *I*
_KATP_: an inward rectifier subunit plus the sulfonylurea receptor. *Science*.

[5] Tucker S. J., Gribble F. M., Zhao C., Trapp S., Ashcroft F. M. (1997). Truncation of Kir6.2 produces ATP-sensitive K^+^ channels in the absence of the sulphonylurea receptor. *Nature*.

[6] Burke M. A., Mutharasan R. K., Ardehali H. (2008). The sulfonylurea receptor, an atypical ATP-binding cassette protein, and its regulation of the KATP channel. *Circulation Research*.

[7] Miki T., Nagashima K., Tashiro F. (1998). Defective insulin secretion and enhanced insulin action in K_ATP_ channel-deficient mice. *Proceedings of the National Academy of Sciences of the United States of America*.

[8] Ashcroft F. M. (2005). ATP-sensitive potassium channelopathies: focus on insulin secretion. *Journal of Clinical Investigation*.

[9] Kane G. C., Liu X.-K., Yamada S., Olson T. M., Terzic A. (2005). Cardiac KATP channels in health and disease. *Journal of Molecular and Cellular Cardiology*.

[10] Cohen M. V., Baines C. P., Downey J. M. (2000). Ischemic preconditioning: from adenosine receptor to KATP channel. *Annual Review of Physiology*.

[11] Cole W. C., McPherson C. D., Sontag D. (1991). ATP-regulated K+ channels protect the myocardium against ischemia/reperfusion damage. *Circulation Research*.

[12] Gross G. J., Auchampach J. A. (1992). Blockade of ATP-sensitive potassium channels prevents myocardial preconditioning in dogs. *Circulation Research*.

[13] Mizumura T., Nithipatikom K., Gross G. J. (1995). Bimakalim, an ATP-sensitive potassium channel opener, mimics the effects of ischemic preconditioning to reduce infarct size, adenosine release, and neutrophil function in dogs. *Circulation*.

[14] Garlid K. D., Dos Santos P., Xie Z.-J., Costa A. D. T., Paucek P. (2003). Mitochondrial potassium transport: the role of the mitochondrial ATP-sensitive K^+^ channel in cardiac function and cardioprotection. *Biochimica et Biophysica Acta (BBA)—Bioenergetics*.

[15] Hodgson D. M., Zingman L. V., Kane G. C. (2003). Cellular remodeling in heart failure disrupts K_ATP_ channel-dependent stress tolerance. *The EMBO Journal*.

[16] Takaba H., Nagao T., Ibayashi S., Kitazono T., Fujii K., Fujishima M. (1996). Altered cerebrovascular response to a potassium channel opener in hypertensive rats. *Hypertension*.

[17] Yuan F., Brandt N. R., Pinto J. M., Wasserlauf B. J., Myerburg R. J., Bassett A. L. (1997). Hypertrophy decreases cardiac K_ATP_ channel responsiveness to exogenous and locally generated (Glycolytic) ATP. *Journal of Molecular and Cellular Cardiology*.

[18] Alvin Z. V., Millis R. M., Hajj-Mousssa W., Haddad G. E. (2011). ATP-sensitive potassium channel currents in eccentrically hypertrophied cardiac myocytes of volume-overloaded rats. *International Journal of Cell Biology*.

[19] Geuns J. M. C. (2003). Stevioside. *Phytochemistry*.

[20] Melis M. S., Sainati A. R. (1991). Participation of prostaglandins in the effect of stevioside on rat renal function and arterial pressure. *Brazilian Journal of Medical and Biological Research*.

[21] Chan P., Xu D.-Y., Liu J.-C. (1998). The effect of stevioside on blood pressure and plasma catecholamines in spontaneously hypertensive rats. *Life Sciences*.

[22] Hsu Y.-H., Liu J.-C., Kao P.-F. (2002). Antihypertensive effect of stevioside in different strains of hypertensive rats. *Chinese Medical Journal*.

[23] Hsieh M.-H., Chan P., Sue Y.-M. (2003). Efficacy and tolerability of oral stevioside in patients with mild essential hypertension: a two-year, randomized, placebo-controlled study. *Clinical Therapeutics*.

[24] Yasukawa K., Kitanaka S., Seo S. (2002). Inhibitory effect of stevioside on tumor promotion by 12-O-tetradecanoylphorbol-13-acetate in two-stage carcinogenesis in mouse skin. *Biological and Pharmaceutical Bulletin*.

[25] Liu J.-C., Kao P.-F., Hsieh M.-H., Chen Y.-J., Chan P. (2001). The antihypertensive effect of stevioside derivative isosteviol in spontaneously hypertensive rats. *Acta Cardiologica Sinica*.

[26] Ma J., Ma Z., Wang J. (2007). Isosteviol reduces plasma glucose levels in the intravenous glucose tolerance test in Zucker diabetic fatty rats. *Diabetes, Obesity and Metabolism*.

[27] Tan W. (2005). The use of kauranes compounds in the manufacture medicament. *PCT Patent*.

[28] Xu D., Zhang S., Foster D., Wang J. (2007). The effects of isosteviol against myocardium injury induced by ischaemia-reperfusion in the isolated guinea pig heart. *Clinical and Experimental Pharmacology and Physiology*.

[29] Xu D., Li Y., Wang J., Davey A. K., Zhang S., Evans A. M. (2007). The cardioprotective effect of isosteviol on rats with heart ischemia-reperfusion injury. *Life Sciences*.

[30] Padrini R., Bova S., Cargnelli G., Piovan D., Ferrari M. (1992). Effects of pinacidil on guinea-pig isolated perfused heart with particular reference to the proarrhythmic effect. *British Journal of Pharmacology*.

[31] Liu Y., Sato T., O'Rourke B., Marban E. (1998). Mitochondrial ATP-dependent potassium channels: novel effectors of cardioprotection?. *Circulation*.

[32] Ježek P., Hlavatá L. (2005). Mitochondria in homeostasis of reactive oxygen species in cell, tissues, and organism. *The International Journal of Biochemistry & Cell Biology*.

[33] Lambert A. J., Brand M. D. (2009). Reactive oxygen species production by mitochondria. *Methods in Molecular Biology*.

[34] Turrens J. F. (2003). Mitochondrial formation of reactive oxygen species. *Journal of Physiology*.

[35] Pain T., Yang X.-M., Critz S. D. (2000). Opening of mitochondrial K(ATP) channels triggers the preconditioned state by generating free radicals. *Circulation Research*.

[36] Cohen M. V., Yang X.-M., Liu G. S., Heusch G., Downey J. M. (2001). Acetylcholine, bradykinin, opioids, and phenylephrine, but not adenosine, trigger preconditioning by generating free radicals and opening mitochondrial K(ATP) channels. *Circulation Research*.

[37] Garlid K. D., Costa A. D. T., Quinlan C. L., Pierre S. V., Dos Santos P. (2009). Cardioprotective signaling to mitochondria. *Journal of Molecular and Cellular Cardiology*.

[38] Sukhodub A., Jovanović S., Qingyou D. U. (2007). AMP-activated protein kinase mediates preconditioning in cardiomyocytes by regulating activity and trafficking of sarcolemmal ATP-sensitive K^+^ channels. *Journal of Cellular Physiology*.

[39] Hu K., Huang C. S., Jan Y. N., Jan L. Y. (2003). ATP-sensitive potassium channel traffic regulation by adenosine and protein kinase C. *Neuron*.

[40] Edwards A. G., Rees M. L., Gioscia R. A. (2009). PKC-permitted elevation of sarcolemmal KATP concentration may explain female-specific resistance to myocardial infarction. *The Journal of Physiology*.

[41] Hanley P. J., Mickel M., Löffler M., Brandt U., Daut J. (2002). KATP channel-independent targets of diazoxide and 5-hydroxydecanoate in the heart. *Journal of Physiology*.

[42] D'hahan N., Moreau C., Prost A. L. (1999). Pharmacological plasticity of cardiac ATP-sensitive potassium channels toward diazoxide revealed by ADP. *Proceedings of the National Academy of Sciences of the United States of America*.

[43] Wojtovich A. P., Brookes P. S. (2009). The complex II inhibitor atpenin A5 protects against cardiac ischemia-reperfusion injury via activation of mitochondrial KATP channels. *Basic Research in Cardiology*.

[44] Wojtovich A. P., Nehrke K. W., Brookes P. S. (2010). The mitochondrial complex II and ATP-sensitive potassium channel interaction: quantitation of the channel in heart mitochondria. *Acta Biochimica Polonica*.

[45] Holmuhamedov E. L., Jahangir A., Oberlin A., Komarov A., Colombini M., Terzic A. (2004). Potassium channel openers are uncoupling protonophores: implication in cardioprotection. *FEBS Letters*.

[46] Bienengraeber M., Alekseev A. E., Abraham M. R. (2000). ATPase activity of the sulfonylurea receptor: a catalytic function for the K_ATP_ channel complex. *The FASEB Journal*.

